# Evaluation of sampling frequency, window size and sensor position for classification of sheep behaviour

**DOI:** 10.1098/rsos.171442

**Published:** 2018-02-07

**Authors:** Emily Walton, Christy Casey, Jurgen Mitsch, Jorge A. Vázquez-Diosdado, Juan Yan, Tania Dottorini, Keith A. Ellis, Anthony Winterlich, Jasmeet Kaler

**Affiliations:** 1 School of Veterinary Medicine and Science, University of Nottingham, Sutton Bonington Campus, Leicestershire LE12 5RD, UK; 2 DXC Technology, Ballybrit Business Park, Galway City H91 WP08, Ireland; 3 Advanced Data Analysis Centre (ADAC), School of Veterinary Medicine and Science, University of Nottingham, Sutton Bonington Campus, Leicestershire LE12 5RD, UK; 4 School of Computer Science, University of Manchester, Manchester M13 9PL, UK; 5 Internet of Things Systems Research, Intel Labs, Leixlip W23 CX68, Ireland

**Keywords:** sheep behaviour, classification algorithm, accelerometer and gyroscope, sensor, signal processing, precision livestock monitoring

## Abstract

Automated behavioural classification and identification through sensors has the potential to improve health and welfare of the animals. Position of a sensor, sampling frequency and window size of segmented signal data has a major impact on classification accuracy in activity recognition and energy needs for the sensor, yet, there are no studies in precision livestock farming that have evaluated the effect of all these factors simultaneously. The aim of this study was to evaluate the effects of position (ear and collar), sampling frequency (8, 16 and 32 Hz) of a triaxial accelerometer and gyroscope sensor and window size (3, 5 and 7 s) on the classification of important behaviours in sheep such as lying, standing and walking. Behaviours were classified using a random forest approach with 44 feature characteristics. The best performance for walking, standing and lying classification in sheep (accuracy 95%, *F*-score 91%–97%) was obtained using combination of 32 Hz, 7 s and 32 Hz, 5 s for both ear and collar sensors, although, results obtained with 16 Hz and 7 s window were comparable with accuracy of 91%–93% and *F*-score 88%–95%. Energy efficiency was best at a 7 s window. This suggests that sampling at 16 Hz with 7 s window will offer benefits in a real-time behavioural monitoring system for sheep due to reduced energy needs.

## 1. Introduction

Recent advances in sensor and smart computing technologies such as global positioning (GPS) trackers, location sensors, proximity loggers, accelerometers, gyroscopes and magnetometers have allowed researchers to study and enrich our understanding of animal movements, space-use patterns, physiology, social interactions and the environment they inhabit [1–4]. These smart technologies have a huge potential to inform resource management and conservation [1,3,4]. One sector that is expected to have a considerable benefit from the use of such technologies is animal welfare and livestock agriculture production [4] as these technologies can give insight into animal behaviour patterns and also allow producers to monitor animals frequently to inform on farm decision making. An accurate and precise behavioural monitoring system could potentially detect behavioural changes correlated to health and welfare status changes in sheep. For example, abnormal postures and the avoidance of specific behaviours can be used to detect foot lesions [5–7]. Similarly, lameness, a widespread welfare problem in sheep farming around the world, will change an animal's normal stance or gait and will also alter behavioural activity [8,9].

Embedded and wearable devices encompassing accelerometer and gyroscope sensors have been used widely to discriminate different behaviours in pets [10] and farm animals [11–15]. Usage of such devices has increased as the technologies have become not only smaller, lighter and cheaper but also capable of processing, exchanging and transmitting an increasingly larger amount of information with a broad range of communication protocols. Such a large amount of data is ideally suited to be processed through machine learning, providing ideal foundations to the development of a new era of intelligent veterinary medicine and science, ultimately contributing to better animal welfare, with significant economic and ecology repercussions. Currently, various machine learning solutions (e.g. support vector machines, decision trees, Kalman filters) have been used on accelerometer data to classify behaviours such as feeding, standing, lying, ruminating, walking and standing in cows [11–13]. In contrast to the large number of automatic behaviour classification studies in cattle, very few studies (with varying results) have been done in sheep using sensor-based technologies [16–18].

There are various factors, such as sampling frequency, window sizes and position of sensor that can affect performance of classification of behaviour using accelerometer and gyroscope data [19]. This is one possible explanation for the varying behaviour classification performance results that have been documented in literature so far for livestock behaviour classification. While there are studies in literature evaluating these aspects for human activity classification with accelerometer data [20–22] there are no studies in precision livestock monitoring that have simultaneously evaluated the impact of these factors on livestock behaviour classification. Often these characteristics are rarely and vaguely described in animal behaviour studies with use of choice of sampling frequency, or position and window size without any robust scientific evaluation.

Moreover, to eventually deliver a complete framework that can efficiently monitor in real time and for long durations, an understanding of the effect of sampling frequencies, sensor positions and window sizes is required. For example, cloud-based systems require real-time streaming of data to a server, which results in a high power drain and reduced battery life [3,23]. An architectural alternative solution to overcome high power demands on the system is the use of embedded based architecture [15,23], where the processing can be performed on the devices attached to the animals. Nevertheless, embedded systems have their own constraints as there is limited processing power and memory on each device. Therefore, to overcome processing power limitations in embedded systems it is necessary to identify methods that optimize the available resources on such devices. A well-informed decision on optimum sampling rate, window sizes and sensor position can result in considerable improvements in energy transmission bandwidth and storage capacity.

The overarching aim of our project is to develop and validate a device that can be used for real-time monitoring and early automatic behaviour classification and lameness in sheep. This study is the first among a series of studies to achieve this. The aim of this study was to evaluate the performance of the random forest algorithm in classifying biologically relevant behaviours in sheep such as lying, standing and walking at three different sampling frequencies (8 Hz, 16 Hz and 32 Hz), three window sizes (3, 5 and 7 s) and two different sensor positions (ear and collar) using tri-axial accelerometer and tri-axial gyroscope data.

## 2. Material and methods

### 2.1. Study site and animals

Before starting the main trial, a pilot study for 2 days was conducted to check the research protocols described below. Ethical permission was obtained for the School of Veterinary Medicine and Science, University of Nottingham. For the main trial in this study, data were collected for 8 days from 5 October 2016 to 7 October 2016 and from 10 October 2016 to 14 October 2016. A total of six sheep were selected via stratified random sampling (age) from a flock of 140 animals at the University of Nottingham. Assessment of body condition score, age and breed was done at day 1. Body condition scoring of sheep is simply a means of assessing the degree of fatness or condition of the living animal and was scored using UK industry guidelines [24,25]. The selected sheep had various body condition scores ranging from 2.5 to 4 and an age ranging from 18 months to 4 years. The breeds of sheep were Texel cross (three individuals), Suffolk cross (one individual) and Mule (two individuals). Sheep were kept in a rectangular 0.3 acre field with a 179.3 m perimeter during the day, when recordings were taking place. At night, sheep were allowed into a larger 2.1 acre field until the next morning's recordings. To facilitate individual identification, sheep were sprayed with coloured livestock spray on either side of the sheep's body with a number between 1 and 19. Numbers sprayed on the sheep were re-sprayed again on day 5 of the trial.

### 2.2. Data collection

Sensor data were collected using a custom-made wearable device based on the Intel® Quark™ SE microcontroller C1000. The device encompassed flash memory, a low-power wide-area radio module and Bosch BMI160 integrated (Bosch-sensortec.com, 2016), ±8*g*, low-power inertial measurement unit (IMU), featuring a 16 bit triaxial gyroscope and a 16 bit triaxial accelerometer. The devices were of dimension 31.6 × 35 × 9 mm and weighed 4 g. The devices were attached to a lightweight Li–Po battery 270 mA h Li-ion battery. The devices where designed for edge-based data processing, classification and reduced transmission. Although at this stage much of the functionality was unutilized and the units where used solely for data acquisition.

**Figure 1.**
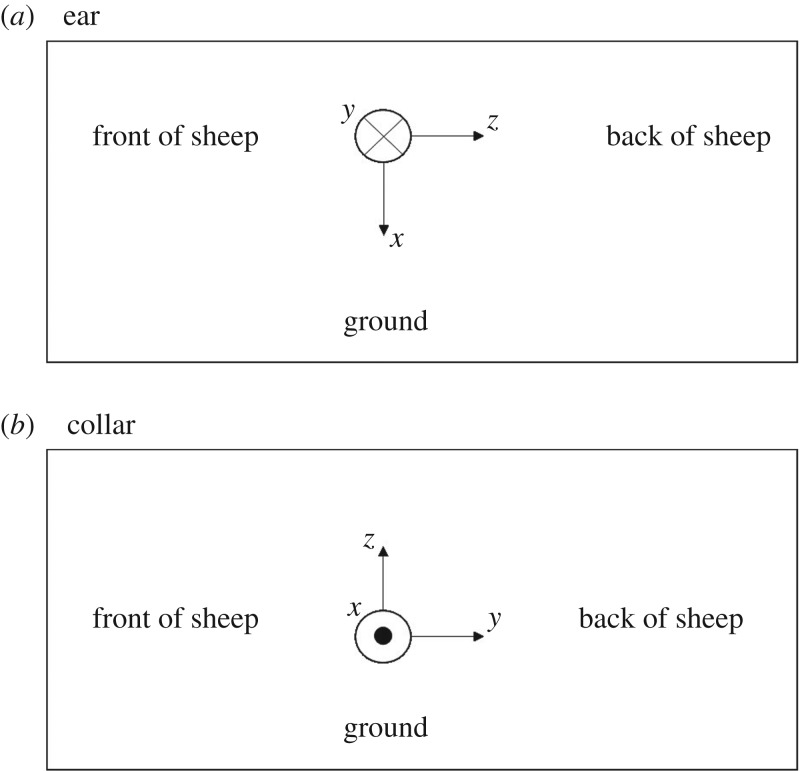
Sensor orientation for ear and collar.

The devices were attached to six sheep at two locations (a) to the existing electronic identification ear tag via a tape and lightweight plastic tie and (b) to neck collar using tape and lightweight plastic tie. All ear-mounted devices were fixed using the orientation illustrated in figure 1*a*, whereas all collar-mounted devices were fixed using the orientation shown in figure 1*b*.

Devices were mounted on sheep at the beginning of each trial day at approximately 09.00 and removed the following morning at 09.00, with the exception Friday, 7 October 2016, when the devices were removed at 16.00 to prevent any possible damage over the weekend when no camera recording took place.

In this study, the device sensors were set to collect data at sampling frequencies of 8, 16 and 32 Hz per axis and each sheep was used for multiple sampling frequencies on different days. Sampling frequencies were set at the beginning of each day, and the setting remained fixed for the entire day of the recording. The devices attached to sheep were set to keep recording raw accelerometer and gyroscope data, with no energy optimization, e.g. sleep features etc. used, until either: the device storage was full or the devices ran out of battery. Different sampling rates had a different drain on battery life [26]. Hence, sampling frequencies of 8, 16 and 32 Hz resulted in raw data with duration of 20, 10 and 5 h, respectively. Each day sensors were prepared by first setting the sampling frequency, then switching them on, while annotating the switching time. Afterwards, to allow time synchronization with the videos, sensors were shaken for 30 s and the start time of the shaking time was annotated. This was followed by a procedure to establish a time reference, where sensors were held horizontally for 30 s and finally held vertically for 30 s. At the end of the 30 s of holding them vertically, the time was annotated. After this procedure, sensors were mounted on sheep. The recorded data were downloaded from the devices.

After removing spurious datasets due to sensor malfunction or other data retrieving issues, a total of 30 datasets (98% of the data collected) from six sheep were used for analysis. Table 1 gives an overview of the total number of datasets collected from ear and collar sensors for different sampling rates.

**Table 1.** Datasets types. Number of successfully extracted datasets from six different sheep for ear and collar sensors according to sampling frequency.

	ear	collar	total
8 Hz	5	5	10
16 Hz	7	7	14
32 Hz	3	3	6

### 2.3. Behavioural observations

Sheep behavioural activities were recorded using a handheld Panasonic HC-V380 video camera with a tripod and were time stamped. The video camera was fitted with a 64 GB SanDisk elite SDXC UHS-1SD card to store the footage. The video camera was set to record in a MP4 50M format with 1080p (1920 × 1080 pixels) quality. With these settings, one SD card could store 3 h and 6 min of footage. Video recording was started each morning immediately after the installation of the first sensor and the starting time of the recording was registered. Video footage was recorded each day in the morning with duration of approximately 2 h. In the recording sessions, the starting and ending times of the recording were annotated.

#### 2.3.1. Behaviours annotation of the videos

Time stamped video recordings of the sheep were processed using the Noldus Observer XT 11(Noldus) (www.noldus.com) software. Coding of the video recordings into the different behavioural categories or classes was performed by playing each video and manually pressing the corresponding code key of the identified behaviour from the set of predefined ones. Behaviours were defined based on the behaviour ethogram developed in a pilot study where sheep were observed and in other literature [18]. Behaviours of interest for this study can be identified according to table 2.

**Table 2.** Definition of sheep behaviours for classification. Different behaviours in sheep used in the classifier according to the ethogram developed.

behaviour states	description
walking	sheep moves forward in a four beat motion for 2 s or more with the head up and orientated in the direction of movement
standing	sheep is standing on their four legs with or without jaw movement, head up or down
lying	sheep lying on ground in sternal or lateral recumbency with or without jaw movement, could be ruminating or in the process of regurgitating a bolus

### 2.4. Data processing

Processing of the data was performed using dedicated software written in Python 3.5 [27], specifically for this project. First, the raw sensor data (accelerometer and gyroscope) and the behaviour information from the video transcripts were aligned using the time stamps. Afterwards each file was discretized into windows of equal length. In this study, window sizes of 3, 5 and 7 s were explored, with a 50% overlap between two consecutive windows [16,19]. During coding of the video recording an individual class label was assigned to each individual data sample. Therefore, the class discretization of each window was determined by looking at the class labels of the individual data samples within each window. If all data samples within a window shared the same activity class, the collective label for the entire window was set to that particular activity class. Windows that contained data points with more than one activity class label were labelled as ‘mixed’ windows and the predominant label was used as the class. The percentage of samples for the ‘mixed’ and ‘non-mixed’ windows in each of the sampling frequencies and window sizes in this study, is shown in table 3.

**Table 3.** Percentage of non-mixed and mixed windows. Summary of the percentage of samples that are non-mixed or mixed for the three different sampling frequencies (8, 16 and 32 Hz) and for the three window sizes (3, 5 and 7 s).

		window size		
sampling frequency	type of sample	3 s	5 s	7 s
8 Hz	non-mixed	97.99	96.85	95.96
	mixed	2.01	3.15	4.04
16 Hz	non-mixed	97.87	96.72	95.55
	mixed	2.13	3.28	4.45
32 Hz	non-mixed	99.40	99.07	98.78
	mixed	0.60	0.93	1.22

For each time window a set of feature characteristics [28] was extracted from the magnitude of the acceleration and the magnitude of the gyroscope which are defined as follows:

$$\begin{array}{rl} \bar{A} & =\sqrt{A_{x}^{2}+A_{y}^{2}+A_{z}^{2}},\\ \bar{G} & =\sqrt{G_{x}^{2}+G_{y}^{2}+G_{z}^{2}}, \end{array}$$

where *A_x_, A_y,_ A_z_*, *G_x_, G_y,_ G_z_* represent the acceleration and gyroscope signals at the axes *x, y, z,* respectively. Eleven different feature characteristics were extracted from both the magnitude of the acceleration and from the magnitude of the gyroscope based on previous literature work [29,30]. In addition, the same feature characteristics were computed from the rate of change of the magnitude of the acceleration and from rate of change of the magnitude of the gyroscope, yielding a total of 44 features that were used in the classification.

Table 4 shows the 11 feature characteristics used for the classification. These include mean, standard deviation, kurtosis, minimum and maximum value [11], interquartile range [31], signal area, absolute signal area, number of zero crossings, dominant frequency [29] and spectral entropy [32].

**Table 4.** Feature characteristics. Feature characteristics computed for each individual window. Here *f* represents the signal and *fs* the sampling frequency.

feature characteristics	description/formula
interquartile range	difference between the 75th percentile and the 25th percentile value of a window
kurtosis	kurtosis calculated from window values
mean	mean of all window values
standard deviation	standard deviation of all window values
minimum value	minimum value of all window values
maximum value	maximum value of all window value
number of zero crossings	number of zero crossings in a window after subtracting the window mean value from every window sample
spectral entropy	power spectral density:
	PSD = |*X*(*f*)^2^|
	*X(f)* – DFT of original signal. Discrete Fourier transform (DFT)
	normalized PSD:
	${\text{PSD}}_{\mathrm{norm}}(f)=\frac{\mathrm{PSD}(f)}{\Sigma^{\mathrm{PSD}}(f)}$
	spectral entropy:
	$\text{SE}=-\Sigma_{\mathrm{norm}}^{\mathrm{PSD}}(f)\cdot \log(\text{PS}{\text{D}}_{\mathrm{norm}}(f))$
dominant frequency	after applying Fourier transformation, this is the frequency at which the signal has its highest power
signal area	signal area:
	$\text{SA}=\Sigma \text{Mag}\cdot \frac{1}{fs}$
	Mag—acceleration or gyroscope magnitude
	*fs*—sampling frequency
absolute signal area	absolute signal area:
	$\text{ASA}=\Sigma |\text{Mag}|\cdot \frac{1}{fs}$

### 2.5. Classification algorithm

Random forests [33,34] are a type of ensemble learning method that is formed through the combination of multiple decision trees trained on the training set. When applied to the test dataset, the predictions of the individual tree models within the random forest are combined into an overall classification decision, e.g. through means of a majority vote or through the application of weights. Because of this, random forest models correct overfitting to the training set and provide a more robust classification performance [35]. A random forest learning algorithm was implemented using Microsoft Azure Machine Learning Studio software [36] and the set of feature characteristics previously described. The random forest algorithm used a resampling bagging method with eight decision trees, 128 random splits per node and minimum of one sample per leaf node.

Data were split into a training set and a test set. The training dataset consisting of 70% of the full dataset was used to develop the model, while the test dataset consisting of the remaining 30% of the original dataset was used for model evaluation and validation [37,38]. The partitioning between training and testing data was carried out using random stratification to ensure that the ratios of the three main activities (walking, standing and lying) remained the same in both datasets.

#### 2.5.1. Performance of the classification

The performance of the random forest classification was evaluated using the following metrics: overall accuracy, precision, recall (also known as sensitivity), *F*-score and specificity, which can be computed as follows:

$$\begin{array}{rl} \text{Overall accuracy} & =\frac{\text{TP}+\text{TN}}{\text{TP}+\text{TN}+\text{FP}+\text{FN}},\\ \text{Precision} & =\frac{\text{TP}}{\text{TP}+\text{FP}},\\ \text{Recall} & =\frac{\text{TP}}{\text{TP}+\text{FN}},\\ F-\text{score} & =2\cdot \frac{\text{precision}\cdot \text{recall}}{\text{precision + recall}}\\ \;\text{and}\;\text{Specificity} & =\frac{\text{TN}}{\text{TN}+\text{FP}}, \end{array}$$

where TP (true positives) is the number of instances where the behaviour of interest (walking, standing or lying) was correctly classified by the algorithm and visually observed. FN (false negatives) is the number of instances where the behaviour of interest was visually observed but was incorrectly classified as some other behaviour by the algorithm. FP (false positives) is the number of instances the behaviour of interest was incorrectly classified by the algorithm but not observed. TN (true negative) is the number of instances where the behaviour of interest was correctly classified as not being observed.

#### 2.5.2. Precision, recall, *F*-score and specificity

A more detailed comparison of the performance of the classification for each individual behaviour can be provided by performance measures such as: precision, recall, *F*-score and specificity, which are computed from the confusion matrix. In this type of matrix, each column represents the behaviour predicted by the classifier while each row represents the observed behaviour. In the electronic supplementary material, we provide the full set of confusion matrices using the random forest algorithm with 3, 5 and 7 s window sizes, for all sampling frequencies (8, 16 and 32 Hz), for both the ear-mounted and collar-mounted sensor.

The overall accuracy represents the total number of correct classifications across all classes. This can be useful when the importance of correctly classifying a specific class is equal for all classes, and provides a meaningful indication of classifier performance, if the dataset is balanced, i.e. all classes are equally represented in the dataset. If one or more classes are of particular interest to the observer (e.g. walking), and the priority is to correctly classify a specific class (possibly at the cost of achieving worse performance for the other classes), precision, recall and specificity give a more in-depth representation of a classifier's performance. The *F*-score gives a measure of a test's accuracy and is a harmonic mean of precision and recall. It reaches its best score at 1 and worst score at 0 [39].

To measure the level of agreement between the classifier based on ear data and the classifier based on collar data, a Cohen's weighted *κ* statistic was conducted [40]. The Cohen's weighted *κ* measure was obtained for the three sampling frequencies (8, 16 and 32 Hz) and the three window sizes (3, 5 and 7 s).

#### 2.5.3. Energy consumption

When considering algorithm implementation, the question of sampling frequency and window size impacting on the energy budget is important, and more specifically what trade-offs if any might be needed in terms of acceptable accuracy versus battery life. Increasing the sampling rate obviously increases the energy required to sample. For example, with respect to the inertia measurement unit (IMU) used and the specific IMU configuration/optimization applied, the difference between 16 and 32 Hz would be approximately 10 µA h versus approximately 17 µA h, or 3.08 years of battery life versus 1.81 years (assuming a 270 mA h battery). So if accuracy at 16 Hz or lower is comparable to 32 Hz there is an obvious gain in using the lower sampling rate.

We calculated the effect on energy consumption when using a 3, 5 and 7 s window size on the device (Intel^®^). The device has two modes of operation ‘raw data capture’ and ‘classification’. When in raw data capture mode the concept of window size does not apply and the device sample rate is constant at the rate chosen. In this mode the device processor persists acquired samples to memory at a constant rate, hence energy consumption is constant. Window size does apply when in classification mode (i.e. classifying using algorithm). When viewed at a window level much is changing, but to more accurately compare windows one must compare across a normalized time span, e.g. on a per hour basis. For example if raw data were captured at 16 Hz, then the input to the classification pipeline remains at 16 Hz and as such its energy consumption is constant (i.e. the IMU energy consumption is the same per hour regardless of window size). Additionally, when viewed on an hourly basis the total bytes pre-processed and classified is constant and so the ‘data processing’ energy consumption is equal. What varies when normalized is the number of times the processor acquires (reads) samples (not the total number of samples) and the number of times it persists (writes) a classification record to memory.

## 3. Results

**Figure 2.**
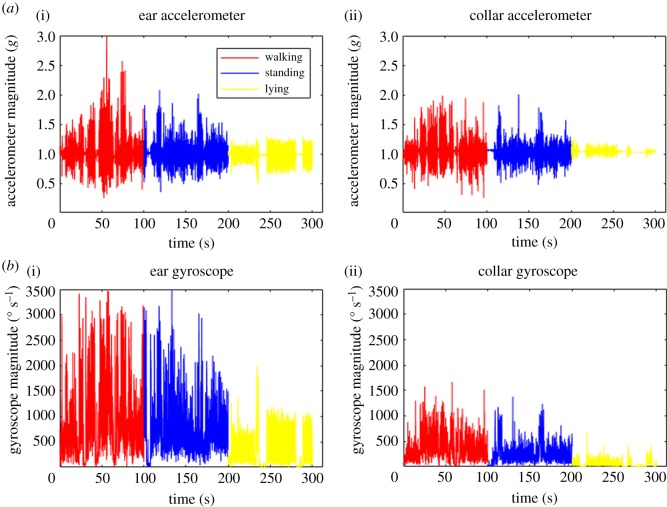
Representative examples of accelerometer and gyroscope data. Accelerometer (*a*) and gyroscope (*b*) data collected for collar and ear mounted sensors with lameness score 0. Colours red, blue and yellow represent walking, standing and lying respectively. Acceleration is in *g* (9.81 m s^−2^) units.

Figure 2 illustrates an example time series of the accelerometer and gyroscope magnitude output for observed periods of lying, standing and walking recorded by ear (figure 2*a*(i),*b*(i)) and collar (figure 2*a*(ii),*b*(ii)) sensors. The sampling frequency for both ear and collar in figure 2 was 16 Hz. From figure 2, it is very clear that is easy to discriminate between lying and any other behaviour due to the associated low overall accelerometer and gyroscope values, for both ear and collar data. Standing and walking behaviours look visually similar, although the walking activity produces higher overall magnitudes for acceleration compared with standing for both collar and ear datasets.

Similar to the observations in the accelerometer data, it is easy to discriminate lying from the other two behaviours using the gyroscope magnitude as they are lower compared with lying and standing (figure 2*b*). Walking also has the highest gyroscope magnitudes as observed in the accelerometer magnitudes. Gyroscope magnitudes for ear data are different to those obtained from collar data, with amplitudes being much larger than their collar counterparts. This is another effect of the much higher overall freedom of movement of the ear sensor compared with the collar sensor. However, the three behaviours are visually distinguishable and consistent with the accelerometer observations.

### 3.1. Assessment of overall classification performance

#### 3.1.1. Overall accuracy

An initial comparison of the performance of the classification across all the window sizes and sampling frequencies can be provided using values of the overall accuracy for both ear- and collar-mounted sensors, as summarized in table 5.

**Table 5.** Overall accuracy. Summary of the overall accuracy metric (in %) for sheep activity classification using both the ear-mounted and collar-mounted sensors, with window sizes of 3, 5 and 7 s with sampling frequencies of 8, 16 and 32 Hz. In bold are the overall accuracies with the highest values when comparing across window sizes. In italics are the overall accuracies with the highest values when comparing only across different sample frequencies.

overall accuracy (%)				
		window size		
sensor position	sampling frequency	3 s	5 s	7 s
ear	8	89	**91**	**91**
	16	88	90	**91**
	32	*94*	***95***	***95***
collar	8	89	**91**	90
	16	90	91	**93**
	32	*94*	***95***	***95***

The highest overall accuracy is 95%, which was obtained for both ear and collar data at 32 Hz with 5 and 7 s windows. The lowest overall accuracy of 89% was obtained for ear data at a sampling frequency of 16 Hz with 3 s window size, and for collar data the lowest accuracy was 89% at a sampling frequency of 8 Hz with 3 s window size. Overall, the accuracies observed were the worst for 3 s windows and best for 7 s windows. For ear data, the differences between 3 and 5 s windows were in a range of 1%–2%, whereas the differences between the 5 and 7 s windows were in a range of 0%–2%. When comparing across different sampling frequencies both ear and collar data gave the best results for 32 Hz data. The average difference between ear and collar accuracy when comparing same window size and sampling rate was very small (0.66%). The average mean accuracy across all sampling frequencies and window sizes in ear data (*µ* = 91.55%, *σ* = 2.55%) was very similar to the average for collar data (*µ* = 92%, *σ* = 2.29%).

#### 3.1.2. Cohen's *κ* measure

Table 6 shows the Cohen's weighted *κ* measure that computes the level of agreement between the accuracy using collar data and the accuracy using ear data [40]. Cohen's weighted *κ* measure was computed using equal weights and using the irr package in R [41]. Cohen's weighted *κ* was computed for each sampling frequency (8, 16 and 32 Hz), and for each window size (3, 5 and 7 s). Overall the *κ*-values computed were worst at 8 Hz and the best at 32 Hz. When comparing across the different window sizes, on average 7 s shows the highest agreement. Except for a combination of 8 Hz and 3 s, all *κ*-values were above 0.80 which represents an almost perfect agreement.

**Table 6.** Weighted Cohen's *κ* measure. Summary of the weighted Cohen's *κ* to measure the level of agreement of the classification between ear and collar data for each combination of sampling frequency and window size. Values between 0.61 and 0.80 represent a substantial agreement whereas values between 0.81 and 0.99 represent an almost perfect alignment.

Cohen's *κ*			
	window size		
sampling frequency	3 s	5 s	7 s
8 Hz	0.799	0.84	0.84
16 Hz	0.883	0.866	0.892
32 Hz	0.882	0.911	0.903

### 3.2. Assessment of the performance of the classification of specific activities

#### 3.2.1. Walking

In table 7, a summary of the precision, recall, *F*-score and specificity is presented. The classification performance for this activity provided the lowest performance values with 80% recall on a 3 s window and 8 Hz sampling frequency for ear and 81% for collar data. Overall, the classification performance for this activity also showed the greatest range of variation with values ranging between 80% (recall) and 99% (specificity) for both ear and 81% (recall) and 99% (specificity) for collar data. Values of *F*-score varied between 83% and 93% for ear data and between 81% and 93% for collar data. The best *F*-score value (93%) for ear data and collar data was obtained for 32 Hz sampling frequency and 5 s window size. Recall values were lowest (80%–92%) for this behaviour but specificity values were very high (95%–99%) for both ear and collar data. Walking behaviour classification for collar data achieved similar performances when compared with standing, with precision ranging from 81% to 94% and recall ranging from 81% to 92%. Performances for this behaviour were comparatively lower when using 8 Hz sample frequency compared with 16 and 32 Hz sample frequencies. Ear and collar data produced similar results for the classification of walking (precision of 88.77%, recall, 87.77%, on average for ear; and precision of 87.77% and recall of 87.55% on average for collar).

**Table 7.** Performance metrics of the classification algorithm. Summary of the precision, recall, *F*-score and specificity metrics for sheep activity classification using both the ear-mounted and collar-mounted sensors with window sizes of 3, 5 and 7 s with sampling frequencies of 8, 16 and 32 Hz. In bold are the highest values for precision, recall, *F*-score and specificity when comparing across window sizes. In italics are the highest values for precision, recall, *F*-score and specificity when comparing only across different sample frequencies.

			window size											
			precision			recall			*F*-score			specificity		
sensor position	behaviour	sampling frequency	3 s	5 s	7 s	3 s	5 s	7 s	3 s	5 s	7 s	3 s	5 s	7 s
ear	walking	8 Hz	86	**90**	88	80	**84**	83	**97**	**97**	**97**	83	**87**	86
		16 Hz	85	86	**88**	83	86	**88**	96	96	**97**	84	86	**88**
		32 Hz	*92*	***93***	*91*	*87*	***92***	*90*	***99***	***99***	*98*	*89*	***93***	*91*
	standing	8 Hz	81	**85**	83	82	85	**86**	94	**96**	95	82	**85**	84
		16 Hz	86	88	**90**	85	86	**89**	93	94	**95**	86	87	**89**
		32 Hz	*92*	***95***	*93*	**93**	*92*	***93***	*96*	***98***	*97*	***93***	***93***	***93***
	lying	8 Hz	94	94	**95**	**96**	**96**	**96**	93	93	**94**	**95**	**95**	**95**
		16 Hz	92	93	**94**	93	94	**95**	94	**95**	**95**	92	**94**	**94**
		32 Hz	*96*	*96*	***97***	*97*	***98***	*97*	*96*	*96*	***97***	***97***	***97***	***97***
collar	walking	8 Hz	81	86	**87**	81	**83**	**83**	95	96	**97**	81	**85**	**85**
		16 Hz	85	88	**89**	89	88	***91***	96	**97**	**97**	87	88	**90**
		32 Hz	*89*	*94*	***91***	*90*	***92***	*91*	*98*	***99***	*98*	*90*	***93***	*91*
	standing	8 Hz	81	**85**	83	79	**84**	**84**	94	**95**	**95**	80	**84**	83
		16 Hz	89	89	**91**	87	90	**91**	***96***	95	**96**	88	90	**91**
		32 Hz	*92*	***93***	***93***	*92*	***93***	***93***	*96*	***97***	***97***	*92*	***93***	***93***
	lying	8 Hz	**95**	**95**	**95**	96	**97**	**97**	**95**	**95**	94	**96**	**96**	**96**
		16 Hz	93	95	**96**	93	94	**95**	95	96	***97***	93	94	**95**
		32 Hz	***97***	***97***	***97***	*97*	***98***	***98***	***97***	***97***	***97***	***97***	***97***	***97***

#### 3.2.2. Standing

The classification performance for this activity was very similar to walking as performance values ranged between 81% and 98% for ear data and 80% and 97% for collar data. Values for the precision, recall and *F*-score varied between 81% and 95% in ear data and between 80% and 93% in collar data. Similarly to walking, the highest performance values were obtained for specificity, with values ranging between 93% and 98% for ear data and 94% to 97% for collar data. For the standing behaviour, precision and recall values were above or very close to 90% for 16 and 32 Hz sample frequencies, and between 81% and 85% for 8 Hz data in both ear and collar data. The classification performance of standing for the ear data (precision of 88.11% and recall of 87.88%, on average) was similar to the collar data (precision of 88.44% and recall of 88.11%, on average).

#### 3.2.3. Lying

For this activity, the best performance results were obtained with values above 93% for precision, recall, *F*-score and specificity in collar data. Additionally, all the performance metrics for the lying behaviour had values above 92% when using ear data. Lying behaviour had the highest values for precision (95.05% on average) and recall (95.94% on average) in both ear and collar data compared with walking (precision 88.27% and recall 86.72%) and standing (precision 88.27% and recall 88%) behaviours. In the lying behaviour, precision and recall values above 92% were observed for any combination of sampling frequency and window size parameters, with a maximum precision of 97% for 32 Hz with a 7 s window in ear data, and 97% maximum precision for 32 Hz with a 3, 5 and 7 s in collar data. A maximum recall of 98% was obtained for 32 Hz data and 5 and 7 s windows using collar data, and a 98% maximum recall for 32 Hz, and 5 s window size for ear data. Of the three different behaviours, lying was the one with the highest overall performance for all the different window sizes and sampling frequencies.

The classification performance for the standing and walking behaviours improved when increasing sampling frequency and window size. Specificity values were in general very high (above 90%) for the different behaviours at all sampling frequencies and window sizes. The highest specificity values were obtained for the walking behaviour (overall average of 97.16%) compared with specificity values of the standing behaviour (overall average of 95.33%) and the lying behaviour (overall average of 95.33%). Specificity values were very similar in ear and collar data; with average values of 97.33%, 95.33% and 94.77% for walking, standing and lying in ear data, and 97%, 95.66% and 95.88% in collar data.

Overall, the best results for ear data are observed when using the 32 Hz sample frequency dataset and 7 s windows, giving an average precision across the three behaviours of 93.66% and an average recall of 93.33%. The best performances for collar sensor data were observed when using the 32 Hz sample frequency and 7 s windows, with an average precision of 93.66% and an average recall of 94% across the three behaviours. Moreover, when comparing between 3 and 5 s window sizes, there is an average increase of 1.5% in accuracy, 2% in precision, 1.77% in recall, 0.61% in *F*-score and 1.77% in specificity. When comparing between 5 and 7 s window sizes, we obtained an average increase of 0.33% in accuracy, 0.444% in recall, 0.111% in *F*-score and 0.056% in specificity and an average 0.056% decrease in precision. There is an average increase of 1.53% on the performance between 3 and 5 s window sizes and an average increase of 0.177% between 5 and 7 s window sizes. The relative difference in performance between 3 and 7 s windows was larger than between 5 and 7 s.

### 3.3. Energy consumption

There were energy consumption benefits for using a 7 s window compared with a 5 or 3 s window, as shown in table 8. Energy consumption benefits included: a reduction of the number of samples acquired for the classification (1200, 720, and 514 for 3, 5 and 7 s, respectively), a reduction of the energy required to acquire the samples (333, 200 and 143 µA h for 3, 5 and 7 s, respectively), and increase of the time before buffer was full to write to flash (96, 160 and 224 s for 3, 5 and 7 s, respectively), a decrease of the number writes per hour (38, 23 and 16 for 3, 5 and 7 s, respectively), and a decrease of the energy used to write to flash (10, 6 and 4 µA h for 3, 5 and 7 s, respectively).

**Table 8.** Energy consumption for data acquisition during classification and written to flash. Measurements of energy are express in µA h (microampere hour). Measurements were provided by Intel®.

	window size		
measure	3 s	5 s	7 s
no. samples/window	48	80	112
no. bytes/window	672	1120	1568
no. sample acquisition/h	1200	720	514
µA h energy (sample acquisitions/h)	333	200	143
no. bytes/h sampled	806 400	806 400	806 400
µA h energy (data processing/h)	1000	1000	1000
SRAM buffer size in bytes	256	256	256
classification record size in bytes	8	8	8
no. seconds before buffer is full and write to flash is executed	96	160	224
no. writes per hour	38	23	16
µA h energy (writes to flash)	10	6	4

## 4. Discussion

To the authors’ knowledge, this is the first study in precision livestock monitoring that simultaneously evaluated effects of position, sampling frequency and window size on behaviour classification. Overall, classification models trained on collar sensor data showed very similar results to models trained on ear data when classifying walking, standing and lying in sheep, with an average difference of ±0.44% in accuracy, in ±0.11% in precision and ±0.74% in recall when comparing across all sampling frequencies and window sizes that were used in this study. A previous study [14] has also shown differences in the performance of a classifier when using sensors in different positions on the body of an animal. The small differences in performance between the collar-mounted sensor and the ear-mounted sensor in our study could be due to the relatively higher noise in the ear data, which results from the greater freedom of movement of the sensor attached to the ear and other behaviours, such as head shaking. In comparison with the neck collar, the advantage of an ear-mounted sensor is that it could potentially be integrated into the current ear tag identifier, providing richer functionality in only one sensor. Whilst the differences between ear and collar position in the current study might be negligible for the classification of behaviours such as standing, walking and lying, the differences could be substantial for other behaviours such as head nodding and grazing/browsing where the accelerometer is likely to register much more weaker-amplitude movements when mounted on the neck. However, this needs to be further investigated.

When comparing across different window sizes, the performance of the random forest algorithm suggests that overall the 7 s window is the best for classifying the sheep behaviours of walking, standing and lying, as in general the accuracy, precision, recall, *F*-score and specificity of the classification increased with increasing window sizes for both ear- and collar-mounted sensors. A recent study on classification of sheep behaviour by Alvarenga *et al*. [18] reported an increase from 82.9% to 83.5% on classification accuracy for activities such as grazing, lying, running, standing and walking when using a 5 s window compared with a 3 s window and a slight decrease to 83.4% when using a 10 s window. When a 14 s window was tried in the current study (results not shown), a significant drop in accuracy was observed (i.e. drop of accuracy to 84% for ear data and 93% for collar data at 32 Hz). The choice of window size depends on the activity to be classified; shorter windows are generally considered better, as they are less likely to have variability or transition in behaviour [20]. At the same time, longer windows tend to be better for more complex behaviours as they are more likely to contain more information and thus result in better classification accuracy [19,20]. Interestingly, in this study the shortest window size (3 s) showed the worst performance for walking and standing behaviours. However, classification performance for lying did not vary much between 3 and 7 s, perhaps due to lying being a comparatively less complex behaviour, thus needing less information for accurate classification. Overall, 7 s window size was also most energy efficient despite the difference in algorithm performance between 7 and 5 s being small. Hence, the trade-off between classification performance and energy consumption is important in the choice of a window size for implementation purposes.

When comparing across the different sampling frequencies for both ear and collar data, there was a general increase in all the performance measures for the sampling frequencies of 16 and 32 Hz. The difference in accuracy between 16 and 32 Hz was small (5% for ear data and 3.33% for collar data, on average). However, increasing the sampling frequency from 16 to 32 Hz can reduce the battery life by up to a half [26]. In other words, an increase up to 5% in classification performance may come at the cost of reducing battery life by up to half. Therefore, the selection of the sampling frequency has to be carefully considered in the configuration of an automated monitoring system as it has a large impact on energy, transmission bandwidth and storage capacity requirements [26].

The classification algorithm in the current study was highly accurate and precise for each of the behaviours as illustrated in tables 5 and 7. The performance results scored much higher than previously reported by other studies on sheep for similar behaviours (e.g. 85.5% accuracy [18] and 87.1% to 89.7% overall accuracy [16]). This could be because in the current study we used features extracted from both accelerometer and gyroscope data for classification unlike previous studies. Lying was the behaviour with the highest precision (95.05% average) and recall (95.94% average) for both ear and collar data when comparing with walking (precision 88.27% and recall 86.72%) and standing (precision 87.94% and recall 88%). Lying can be more easily classified as sheep move significantly less than when standing and walking. The highest misclassification was observed between walking and standing (see confusion matrices in the electronic supplementary material). This is probably due to the grazing activity (which is most likely to occur when sheep are standing) causing accelerations similar to walking, leading to misclassifications. In the current study, grazing activity was not separately recorded and further work is needed to investigate grazing and non-grazing activities.

The computational cost of the algorithm and its feasibility to be implemented on the device is another factor that has to be considered when developing a behavioural monitoring system. We chose a random forest algorithm to implement the classifier in the current study as random forest has low computational costs. Previous studies have used complex methods such as hidden Markov models (HMM) [13], support vector machines (SVM) [11] and generalized mixed linear models (GMLM) [12] or simple methods such as decision trees [13] for the classification of behaviours in farm animals. HMM, SVM and GMLM can have high computational cost and hence they are typically less appealing for automated, real-time monitoring devices. Decision trees can offer a low computational cost; however, they tend to overfit the data [2]. Random forest algorithms can improve the overfitting problem of decision trees algorithms while maintaining a relatively low computational cost [2]. Hence, random forest represents a potentially good candidate for embedding in sensors mounted on animals. Additionally, the results obtained in this study show that a random forest classifier can accurately classify different relevant behaviours in sheep with a very high accuracy.

While in the study we have evaluated different window sizes and sampling frequencies that are suited for the real-time monitoring of behaviour classification of sheep; we need to validate our results in a field trial. In addition, we did not evaluate feature importance and further selection which will be important in terms of computational cost for real-time monitoring.

## 5. Conclusion

The results from this study show that biologically relevant behaviours in sheep such as walking, standing and lying, can be accurately classified (89–95%), using a random forest classifier and extract specific feature characteristics that consider window size, position and sampling frequency. Evaluation of window sizes suggests 7 s to be the best window size for achieving high classification accuracy of walking, standing and lying and also with respect to energy performance. The highest performance of the classifier was obtained when using a sampling frequency of 32 Hz. However, results using a 16 Hz sample frequency were comparable, thus suggesting significant benefits of using 16 Hz for real-time monitoring. Overall, the level of agreement of the accuracy of the classification between collar and ear data was high (0.84–0.911), except at 8 Hz and 3 s. However, ear-mounted sensors could be more easily integrated into the already existing ear tag identifiers for sheep.

## Supplementary Material

Supporting documents

## Data accessibility

All data files, can be found at Dryad: http://dx.doi.org/10.5061/dryad.h5c80 [42].

## References

[1] McGowan J et al. 2016 Integrating research using animal-borne telemetry with the needs of conservation management. J. Appl. Ecol. 54, 423–429. (doi:10.1111/1365-2664.12755)

[2] Valleta JJ, Torney C, Kings M, Thornton A, Madden J 2017 Applications of machine learning in animal behaviour studies. Anim. Behav. 124, 203–220. (doi:10.1016/j.anbehav.2016.12.005)

[3] Neethirajan S 2017 Recent advances in wearable sensors for animal health management. Sens. Bio Sens. Res. 12, 15–29. (doi:10.1016/j.sbsr.2016.11.004)

[4] Jukan A, Masip-Bruin X, Amla N 2017 Smart computing and sensing technologies for animal welfare: a systematic review. ACM Comput. Surveys 50, Article No. 10 (doi:10.1145/3041960)

[5] Molony V, Kent JE 1997 Assessment of acute pain in farm animals using behavioural and physiological measurements. J. Anim. Sci. 75, 266–272. (doi:10.2527/1997.751266x) 9027575 10.2527/1997.751266x

[6] Fitzpatric J, Scott M, Nolan A 2006 Assessment of pain and welfare on sheep. Small Ruminant Res. 62, 55–61. (doi:10.1016/j.smallrumres.2005.07.028)

[7] Ley SJ, Livingston A, Waterman AE 1989 The effect of chronic clinical pain on thermal mechanical thresholds in sheep. Pain 39, 353–357. (doi:10.1016/0304-3959(89)90049-3) 2616185 10.1016/0304-3959(89)90049-3

[8] Kaler J, Wassink GJ, Green LE 2009 The inter- and intra-observer reliability of locomotion scoring scale for sheep. Vet. J. 180, 189–194. (doi:10.1016/j.tvjl.2007.12.028) 18308594 10.1016/j.tvjl.2007.12.028

[9] Miedema JM 2010 Investigating the use of behavioural, accelerometer and heart rate measurements to predict calving in dairy cows. PhD thesis, Royal (Dick) School of Veterinary Studies, The University of Edinburgh.

[10] Gerencsér L, Vásárhelyi G, Nagy M, Vicsek T, Miklósi A 2013 Identification of behaviour in freely moving dogs (*Canis familiaris*) using inertial sensors. PLoS ONE 8, e0077814 (doi:10.1371/journal.pone.0077814) 10.1371/journal.pone.0077814 PMC3820959 24250745

[11] Martiskainen P, Järvinen M, Skön JP, Tiirikainen J, Kolehmainen M, Mononen J 2009 Cow behaviour pattern recognition using a three-dimensional accelerometer and support vector machines. Appl. Anim. Behav. Sci. 119, 32–38. (doi:10.1016/j.applanim.2009.03.005)

[12] Robert B, White BJ, Renter DG, Larson RL 2009 Evaluation of three-dimensional accelerometers to monitor and classify behaviour patterns in cattle. Comput. Electron. Agric. 67, 80–84. (doi:1016/j.compag.2009.03.002)

[13] Vázquez-Diosdado JA, Barker ZE, Hodges HR, Amory JR, Croft DP, Bell NJ, Codling EA 2015 Classification of behaviour in housed dairy cows using an accelerometer-based activity monitoring system. Anim. Biotelem. 3, 15 (doi:1186/s40317-015-0045-8)

[14] Moreau M, Siebert S, Buerkert A, Schlecht E 2009 Using of a tri-axial accelerometer for automated recording and classification of goats' grazing behaviour. Appl. Anim. Behav. Sci. 199, 158–170. (doi:1016/j.applanim.2009.04.008)

[15] Marchioroa GF, Cornou C, Kristensen AR, Madsen J 2011 Sows’ activity classification device using acceleration data – a resource constrained approach. Comput. Electron Agr. 77, 110–117. (doi:1016/j.compag.2011.04.004)

[16] Marais J, Le Roux SP, Wolhuter R, Niesler T 2014 Automatic classification of sheep behaviour using 3-axis accelerometer data. In Proc. 25th Annual Symp. Pattern Recognition Association of South Africa (PRASA), Cape Town, South Africa, 27–28 November, pp. 97–102.

[17] Radeski M, Ilieski V 2016 Gait and posture discrimination in sheep using a tri-axial accelerometer. Animal 11, 1249–1257. (doi:10.1017/S175173111600255X) 27903315 10.1017/S175173111600255X

[18] Alvarenga FAP, Borges I, Palković RJ, Oddy VH, Dobos RC 2016 Using a three-axis accelerometer to identify and classify sheep behaviour at pasture. Appl. Anim. Behav. Sci. 181, 91–99. (doi:10.1016/j.applanim.2016.05.026)

[19] Bersch SD, Azzi D, Khusainov R, Achumba IW, Ries J 2014 Sensor data acquisition and processing parameter for human activity classification. Sensors 14, 4239–4270. (doi:10.3390/s140304239) 24599189 10.3390/s140304239 PMC4003942

[20] Banos O, Galvez J-M, Damas M, Pomares H, Rojas I 2014 Window size impact in human activity recognition. Sensors 14, 6474–6499. (doi:10.3390/s140406474) 24721766 10.3390/s140406474 PMC4029702

[21] Gao L, Bourke AK, Nelson J 2014 Evaluation of accelerometer based multi-sensor versus single-sensor activity recognition systems. Med. Eng. Phys. 36, 779–785. (doi:10.1016/j.medengphy.2014.02.012) 24636448 10.1016/j.medengphy.2014.02.012

[22] Attal F, Mohammed S, Dedabrishvii M, Chamroukhi F, Oukhellou L, Amirat Y 2015 Physical human activity recognition using wearable sensors. Sensors 15, 31 314–31 338. (doi:10.3390/s151229858) 10.3390/s151229858 PMC4721778 26690450

[23] Ohja T, Misra S, Raghuwanshi NS 2015 Wireless sensor network for agriculture: the state-of-the-art in practice and future challenges. Comput. Electron. Agr 118, 66–84. (doi:10.1016/j.compag.2015.08.011)

[24] Russel A 1984 Body condition scoring of sheep. Practice 6, 91–93. (doi:10.1136/inpract.6.3.91) 10.1136/inpract.6.3.91 6735487

[25] Agriculture and Horticulture Development Board (AHDB). 2017 See https://beefandlamb.ahdb.org.uk/wp-content/uploads/2013/06/brp_l_Sheep_BCS_190713.pdf.

[26] Khan A, Hammerla N, Mellor S, Plötz T 2016 Optimising sampling rates for accelerometer-based human activity. Pattern Recogn. Lett. 73, 33–40. (doi:10.1016/j.patrec.2016.01.001)

[27] Python Software Foundation. 2010 *Python Language Reference, version 2.7*. See http://www.python.org.

[28] Qasem L, Cardew A, Wilson A, Griffiths I, Halsey LG, Shepard ELC, Gleiss AC, Wilson R 2012 Tri-axial dynamic acceleration as a proxy for animal energy expenditure; should we be summing values or calculating the vector? PLoS ONE 7, e31187 (doi:10.1371/journal.pone.0031187) 22363576 10.1371/journal.pone.0031187 PMC3281952

[29] Figo D, Diniz PC, Ferreira DR, Cardoso JM 2010 Preprocessing techniques for context recognition from accelerometer data. Pers. Ubiquit. Comput. 14, 645–662. (doi:10.1007/s00779-010-0293-9)

[30] Piercee SJ, Goulermas JY, Kenney PJ, Howard D 2009 A comparison of feature extraction methods for classification of dynamic activities from accelerometer data. IEEE. T. Bio. Med. Eng. 56, 871–879. (doi:10.1109/TBME.2008.2006190) 10.1109/TBME.2008.2006190 19272902

[31] Wundersitz DW, Josman C, Gupta R, Netto KJ, Gastin PB, Robertson S 2015 Classification of team sport activities using a single wearable tracking device. J. Biomech. 48, 3975–3981. (doi:10.1016/j.biomech.2015.09.015) 26472301 10.1016/j.jbiomech.2015.09.015

[32] Bao L, Intille SS. 2004 Activity recognition from user-annotated acceleration data. In Inter. Conf. Pervasive Comput, Linz and Vienna, Austria, 21–23 April, pp. 1–17. Berlin, Germany: Springer.

[33] Breiman L 2001 Random forests. Mach. Learn. 45, 5–32. (doi:10.1023/A:10109334)

[34] Cutler DR, Edwards TC, Beard KH, Cutler A, Hess KT, Gibson J, Lawler JJ 2007 Random forest for classification in ecology. Ecology 88, 2783–2792. (doi:10.1890/07-0539.1) 18051647 10.1890/07-0539.1

[35] Hastie T, Tibshirani R, Friedman J 2008 The elements of statistical learning, 2nd edn Berlin, Germany: Springer.

[36] Fontama V, Barga R, Tok WH 2015 Predictive analytics with Microsoft Azure machine learning: build and deploy actionable solutions in minutes. Berkeley, CA: Apress.

[37] Browne MW 2000 Cross-validation methods. J. Math. Psychol. 44, 108–132. (doi:10.1006/jmps.1999.1279) 10733860 10.1006/jmps.1999.1279

[38] Burman P 1989 A comparative study of ordinary cross-validation, v-fold cross-validation and the repeated learning-testing methods. Biometrika 76, 503–514. (doi:10.2307/2336116)

[39] Mitchell E, Monaghan D, O'Connor NE 2013 Classification of sporting activities using smartphone accelerometers. Sensors 13, 5317–5337. (doi:10.3390/s130405317) 23604031 10.3390/s130405317 PMC3673139

[40] Ben-David A 2008 Comparison of classification accuracy using Cohen's weighted kappa. Expert Syst. Appl. 34, 825–832. (doi:10.1016/j.eswa.2006.10.022)

[41] R Core Team. 2013 R: A language and environment for statistical computing. Vienna, Austria: R Foundation for Statistical Computing See https://www.R-project.org/.

[42] Walton E, Casey C, Mitsch J, Vázquez-Diosdado JA, Yan J, Dottorini T, Ellis KA, Winterlich A, Kaler J 2018 Data from: Evaluation of sampling frequency, window size and sensor position for classification of sheep behaviour Dryad Digital Repository. (doi:10.5061/dryad.h5c80) 10.1098/rsos.171442 PMC5830751 29515862

