# Supplementary material for: Evaluation of sampling frequency, window size and sensor position for classification of sheep behaviour
Source: R Soc Open Sci. 2018 Feb 7;5(2):171442. doi: 10.1098/rsos.171442 (PMC5830751; doi:10.1098/rsos.171442)
Supplement: Supporting documents [file rsos171442supp1.docx]

**Supporting documents**

**S1 File. Tables for the confusion matrix across all parameters.**

|  | | | **3s** | | | |
| --- | --- | --- | --- | --- | --- | --- |
|  |  |  |  | **Predicted Class** | | |
|  |  |  |  | **Walking** | **Standing** | **Lying** |
| **Actual Class** | **8 Hz** | **Ear** | **Walking** | 1030 | 200 | 63 |
|  |  |  | **Standing** | 125 | 1290 | 167 |
|  |  |  | **Lying** | 31 | 105 | 3216 |
|  |  | **Collar** | **Walking** | 1034 | 218 | 41 |
|  |  |  | **Standing** | 187 | 1264 | 131 |
|  |  |  | **Lying** | 27 | 77 | 3248 |
|  | **16 Hz** | **Ear** | **Walking** | 1373 | 184 | 60 |
|  |  |  | **Standing** | 207 | 2298 | 184 |
|  |  |  | **Lying** | 66 | 158 | 2971 |
|  |  | **Collar** | **Walking** | 1430 | 148 | 39 |
|  |  |  | **Standing** | 156 | 2371 | 162 |
|  |  |  | **Lying** | 65 | 149 | 2981 |
|  | **32 Hz** | **Ear** | **Walking** | 638 | 62 | 19 |
|  |  |  | **Standing** | 56 | 1358 | 51 |
|  |  |  | **Lying** | 5 | 50 | 2145 |
|  |  | **Collar** | **Walking** | 646 | 55 | 18 |
|  |  |  | **Standing** | 68 | 1350 | 47 |
|  |  |  | **Lying** | 11 | 36 | 2153 |
|  |  |  |  |  |  |  |
|  | | | **5s** | | | |
|  |  |  |  | **Predicted Class** | | |
|  |  |  |  | **Walking** | **Standing** | **Lying** |
| **Actual Class** | **8 Hz** | **Ear** | **Walking** | 647 | 85 | 34 |
|  |  |  | **Standing** | 53 | 782 | 86 |
|  |  |  | **Lying** | 31 | 42 | 1931 |
|  |  | **Collar** | **Walking** | 647 | 99 | 20 |
|  |  |  | **Standing** | 101 | 751 | 69 |
|  |  |  | **Lying** | 18 | 50 | 1936 |
|  | **16 Hz** | **Ear** | **Walking** | 831 | 66 | 24 |
|  |  |  | **Standing** | 72 | 1370 | 85 |
|  |  |  | **Lying** | 30 | 75 | 1738 |
|  |  | **Collar** | **Walking** | 844 | 54 | 23 |
|  |  |  | **Standing** | 79 | 1387 | 61 |
|  |  |  | **Lying** | 34 | 59 | 1750 |
|  | **32 Hz** | **Ear** | **Walking** | 386 | 37 | 6 |
|  |  |  | **Standing** | 29 | 802 | 43 |
|  |  |  | **Lying** | 5 | 21 | 1292 |
|  |  | **Collar** | **Walking** | 392 | 35 | 2 |
|  |  |  | **Standing** | 27 | 808 | 39 |
|  |  |  | **Lying** | 7 | 29 | 1282 |
|  |  |  |  |  |  |  |
|  |  |  |  |  |  |  |
|  | | | **7s** | | | |
|  |  |  |  | **Predicted Class** | | |
|  |  |  |  | **Walking** | **Standing** | **Lying** |
| **Actual Class** | **8 Hz** | **Ear** | **Walking** | 480 | 44 | 17 |
|  |  |  | **Standing** | 30 | 563 | 50 |
|  |  |  | **Lying** | 19 | 29 | 1379 |
|  |  | **Collar** | **Walking** | 477 | 48 | 16 |
|  |  |  | **Standing** | 48 | 553 | 42 |
|  |  |  | **Lying** | 15 | 31 | 1381 |
|  | **16 Hz** | **Ear** | **Walking** | 600 | 44 | 18 |
|  |  |  | **Standing** | 56 | 995 | 49 |
|  |  |  | **Lying** | 26 | 64 | 1251 |
|  |  | **Collar** | **Walking** | 619 | 27 | 16 |
|  |  |  | **Standing** | 48 | 1003 | 49 |
|  |  |  | **Lying** | 21 | 66 | 1254 |
|  | **32 Hz** | **Ear** | **Walking** | 281 | 22 | 2 |
|  |  |  | **Standing** | 15 | 571 | 35 |
|  |  |  | **Lying** | 4 | 15 | 922 |
|  |  | **Collar** | **Walking** | 278 | 22 | 5 |
|  |  |  | **Standing** | 13 | 584 | 24 |
|  |  |  | **Lying** | 5 | 16 | 920 |

Confusion matrices using 3s, 5s and 7s window sizes and 8Hz, 16 Hz and 32 Hz sampling rates for both ear mounted and collar mounted sensors.
